# Physical Activity and Acute Care Utilization in Older Adults Living with Mild Cognitive Impairment and Dementia in the Upper Midwest of the United States

**DOI:** 10.1002/alz.086925

**Published:** 2025-01-09

**Authors:** Mairead M. Bartley, Jennifer St. Sauver, Darrell R. Schroeder, Nandita Khera, Emma Fortune, Joan M. Griffin

**Affiliations:** ^1^ Mayo Clinic, Rochester, MN USA; ^2^ Mayo Clinic, Phoenix, AZ USA

## Abstract

**Background:**

People living with dementia have high rates of emergency department (ED) use and hospitalizations. Identifying factors that influence acute care utilization is important. We examined the influence of physical activity levels on risk of hospitalization and emergency department (ED) use in a population of older people living with mild cognitive impairment (MCI) or dementia.

**Methods:**

We included people with a diagnosis of MCI or dementia followed in Community Internal Medicine at Mayo Clinic, age 55 years and older, who had a clinic visit between June 1, 2019 and June 30, 2021 and had completed a social determinants of health questionnaire about physical activity levels. Physical activity was classified based on responses to questions about time spent exercising per week and rated as sufficiently active (≥150 minutes per week), insufficiently active (10‐140 minutes) or physically inactive (0 minutes). The risk of hospitalization and ED visits across physical activity levels was examined using quasi‐Poisson regression and presented as rate ratios (95% confidence interval (CI)), with adjustment for demographics, Charlson Comorbidity Index, marital status, living arrangement and body mass index.

**Results:**

A total of 3090 persons living with MCI (n = 1308) or dementia (n = 1782) were included. People who were physically inactive were more likely to be older, female, not married, obese, have higher comorbid burden and a dementia diagnosis (p<0.001). Hospitalization and ED visit rates in those who were physically inactive were approximately double the rates for the sufficiently active group (p<0.001). Compared with those who were sufficiently active, people who were physically inactive had a significantly higher risk of hospitalization (rate ratio 1.73, 95% CI 1.30‐2.31) and ED visits (rate ratio 1.59, 95% CI 1.26‐2.01).

**Conclusions:**

Physical inactivity in older people living with MCI or dementia is associated with an increased risk of ED visits and hospitalization. Targeted physical activity interventions could potentially impact on acute care utilization in this population. Our findings emphasize an area for public health promotion in people living with MCI or dementia.

